# Clinical and Epidemiological Factors Associated with Mortality in Parkinson's Disease in a Brazilian Cohort

**DOI:** 10.1155/2015/959304

**Published:** 2015-12-27

**Authors:** Gustavo Costa Fernandes, Mariana Peixoto Socal, Artur Francisco Schumacher Schuh, Carlos R. M. Rieder

**Affiliations:** ^1^Federal University of Rio Grande do Sul, 90040060 Porto Alegre, RS, Brazil; ^2^Hospital Moinhos de Vento, 90035001 Porto Alegre, RS, Brazil; ^3^Johns Hopkins Bloomberg School of Public Health, Baltimore, MD 21205, USA; ^4^Hospital de Clínicas de Porto Alegre, 90035903 Porto Alegre, RS, Brazil

## Abstract

*Background*. Prognosis of PD is variable. Most studies show higher mortality rates in PD patients compared to the general population. Clinical and epidemiologic factors predicting mortality are poorly understood.* Methods*. Clinical and epidemiologic features including patient history and physical, functional, and cognitive scores were collected from a hospital-based cohort of PD patients using standardized protocols and clinical scales. Data on comorbidities and mortality were collected on follow-up.* Results*. During a mean follow-up of 4.71 years (range 1–10), 43 (20.9%) of the 206 patients died. Those who died had higher mean age at disease onset than those still alive at the last follow-up (67.7 years versus 56.3 years; *p* < 0.01). In the univariate analysis, age at baseline was associated with decreased survival. In the adjusted Cox proportional hazards model, age at disease onset and race/ethnicity were predictors of mortality.* Conclusions*. Late age at disease onset and advanced chronological age are associated with decreased survival. Comorbidities and PD characteristics were not associated with decreased survival in our sample. Race/ethnicity was found in our study to be associated with increased hazard of mortality. Our findings indicate the importance of studying survival among different populations of PD patients.

## 1. Introduction

Parkinson's disease has a broad range of clinical features, with great variability between individuals. Overall prognosis is difficult to establish due to the heterogeneity of data in the literature. Cohort studies tend to show increased mortality as compared to the general population [1]. However, results are varied, mostly due to study designs, diagnostic criteria, and outcome measures. One study showed similar mortality rates between PD patients and the UK population [2]. A number of clinical and epidemiological factors have been shown to increase the risk of death in PD patients, such as age [1], motor symptoms [2–5], age at disease onset [4, 6], and presence of dementia [7, 8]. Prognostication has relevance not only in clinical practice, influencing long-term care planning, but also in research settings. Clinical trials should be able to study homogeneous populations, therefore providing results that are more accurate.

A Brazilian community-based survey showed a PD prevalence of 3.3% in individuals over the age of 64 years [9], one of the highest in recent studies. Evaluation of factors influencing mortality is necessary in different populations, given the heterogeneity of the disorder. In addition, the impact of health care is an important contributor to prognosis. Access to specialized neurological care and specific drugs may be problematic in some countries, influencing mortality in different settings. These data are currently lacking in our population. Given its importance to give input to specific studies such as cost-effectiveness analyses of Parkinson's treatments in local settings and serve as a comparator to future studies, we aimed to identify these factors.

## 2. Methods

A cohort consisting of 233 patients was followed with regular visits at a specialized Movement Disorders Clinic in Porto Alegre, Brazil, a tertiary referral center in a region of 10 million inhabitants. Recruitment began in 2006 and ended in 2012, including all patients with a clinical diagnosis of PD. All patients were examined by at least one movement disorder specialist and fulfilled the United Kingdom Parkinson's Disease Society Brain Bank criteria for idiopathic PD [10]. There were no exclusion criteria other than not meeting the diagnosis requirement. On the first evaluation, data on epidemiological and clinical features were collected using a standardized protocol and clinical scales. Disease severity was scored according to Hoehn&Yahr (H&Y) scale and disability was measured with the Schwab-England Activities of Daily Living scale. Motor features were evaluated using the Unified Parkinson's Disease Rating Scale (UPDRS) and cognitive status was assessed using the Folstein Minimental State Examination. Diagnosis of depression was based on clinical assessment using DSM-IV criteria. Age at onset of PD was defined as the age at which the first symptom was noticed by the patient. Although relying on self-reported information on symptom onset may carry the risk of recollection bias, many patients presented to us with long standing symptoms without a previous diagnosis, so duration of disease would be underestimated. Patients were classified into late-onset (>60 years) and nonlate-onset (≤60 years).

Motor subtype was defined using a score based on the UPDRS, previously described in the DATATOP study [11]. Patients were divided into tremor predominant subtype and postural instability/gait disorder (PIGD) subtype. The tremor score was calculated as the mean of the following items: right and left arm tremor by history, tremor at rest (face, lips, chin, and limbs), and postural or action tremor by examination. The PIGD features were determined using the mean score of the following items: falling, freezing, and walking difficulty by history and gait and postural instability by examination. Race/ethnicity was divided into white and nonwhite individuals (which included blacks, Asians, and Indians. Together they comprised 11% of the study sample). Cognitive status was defined as “demented” and “nondemented” using a cut-off score of ≤23 for the former (maximum: 30) in the Minimental State Exam, which showed good sensitivity and specificity as a screening tool in our population [12].

Reassessments, including motor examination and UPDRS scores, were performed from January until August 2014. Medical records of all patients who failed visits within the last 12 months were reviewed and data on mortality, including year and cause of death, were collected from medical records and telephone calls to family members. Comorbidities were recorded at baseline and again at reassessment. Comorbid conditions of those who failed visits were reassessed by telephone call using a standardized questionnaire based on previous studies [13, 14].

The study was approved by the ethical committee of the Hospital de Clínicas de Porto Alegre. Written informed consents were obtained from all patients at baseline. A new informed consent was provided by patients or by family members of those who died.

## 3. Statistical Analysis

Exploratory data analysis was conducted via Student's *t*-test, Chi-square test, and ANOVA according to each variable's characteristics. Kaplan-Meier curves were drawn in order to explore survival patterns in the entire sample and between selected groups. Cox proportional hazards models were employed to identify factors associated with decreased survival. Time from symptom onset was used as the time scale. Proportionality of hazards was tested using Schoenfeld residuals. Time-varying components were added to the model for the variables whose Schoenfeld residuals tests did not indicate proportionality of hazards. The adjusted model included all listed covariates simultaneously. Analysis was performed using the software STATA version 12 (StataCorp LP, College Station, TX, USA).

## 4. Results

Table 1 displays the baseline characteristics of the study population. Two hundred and thirty-three patients were evaluated at baseline. Twenty-seven (11.6%) patients were lost to follow-up. During the average follow-up of 4.71 years (range 1–10), 43 (20.9%) of the 206 remaining patients died.

Hypertension, smoking, and heart disease were the most frequent comorbidities reported (Table 2). Only hypertension was significantly different between the groups, being less prevalent in individuals who died. Depression was diagnosed in 31% of all patients, with no difference between those still alive and those who died. There was no difference on Hoehn&Yahr score and Schwab-England scale between groups at baseline.

Mean age at disease onset was 57.9 (SD 11.5) years. Mean age at death was 75.5 (SD 9.3) years. Those who died had a significantly higher mean age at onset than those still alive at last follow-up (67.7 years versus 56.3 years, *p* < 0.01). Average disease duration until death was 11.8 (SD 5.12) years. Median survival time was 12 years, within a range of 4–28 years. Patients with age at disease onset <60 years had a median survival time of 14 years and patients with disease onset ≥60 years had a median survival time of 11 years (*p* < 0.01). Main cause of death was pneumonia (28%), followed by cardiovascular diseases (19%) and cancer (14%) (Table 3).

Women were found to have a higher hazard ratio at baseline compared to men. However, when we allow the effect of gender on survival to vary over time, we find that, after year 12, the relationship gets inverted and males are exposed to a higher hazard than females (see supplementary Figure  1 in Supplementary Material available online at http://dx.doi.org/10.1155/2015/959304). This finding may be affected by the low number of patients with disease duration over 12 years in our sample.

In the adjusted Cox proportional hazards model, main predictors of mortality were age at disease onset (HR 1.06, 95% CI 1.02–1.10, per year increase, and *p* = 0.004) and nonwhite race (HZ 3.41, 95% CI 1.21–9.58, and *p* = 0.021) (Table 4). Higher age at first visit was significantly associated with increased risk of death in the univariate analysis model (HR 1.05, 95% CI 1.02–1.08, per year increase, and *p* = 0.001), while age at onset <60 years was associated with increased chance of survival (HR 0.32, 95% CI 0.16–0.64, and *p* = 0.001). However, both could not be included in the adjusted model because of collinearity. In the univariate analysis, dementia was associated with increased mortality (HR 1.99, 95% CI 1.01–3.92, and *p* = 0.046) but did not reach statistical significance in the adjusted model. Motor subtypes were not associated with risk of mortality in any model.

## 5. Discussion

There is an important interindividual heterogeneity in the clinical course of PD. One problem concerning studies in PD patients is diagnostic accuracy. We used strict diagnostic criteria, in accordance with the UK Parkinson's Disease Society Brain Bank, which was shown to have a sensitivity of 91% and a positive predictive value of up to 98% [15]. Furthermore, diagnostic accuracy significantly increases with disease duration, as clinical features become more evident [16]. Since patients were followed up with regular visits and examined by movement disorders specialists, we were able to detect potential misdiagnosis, thus reducing the chance of selection bias.

Mean age at onset of symptoms was 57.9 (SD 11.5) years, somewhat lower than previous studies. A recent analysis of 30 years of incident PD cases detected a median age at onset of 63 years [17]. Our study showed a median survival time of 12 years from disease onset. However, there was a wide range from 4 to 28 years, highlighting the great variability of prognosis. This important feature has been shown in previous studies [4, 18, 19].

As others have reported [18, 20], pneumonia was the main cause of death (28%). Cardiovascular diseases (19%) and cancer (14%) followed. Some population-based studies have demonstrated a higher rate of pneumonia as the cause of death compared to the general population and a lower rate of death due to cardiovascular causes [19, 21]. It is clear now that pneumonia and infectious diseases in general are the main cause of death in PD, probably associated with immobility and dysphagia.

Clinical comorbidities did not influence significantly the risk of death, in accordance with a community-based study specifically approaching this subject [22]. Dementia was associated with increased risk of mortality in the univariate analysis (HR 1.99, 95% CI 1.01–3.92, and *p* = 0.046), but not in the adjusted model. However, cognitive impairment and dementia seem to be well-established risk factors for mortality in PD patients [3–5]. Since cognitive impairment and dementia are known to increase with age, this result may be due to insufficient sample and/or to short follow-up time.

Race/ethnicity was found to be an important predictor of mortality, with nonwhite individuals having an increased risk of death. However, interpretation of this finding is very complex. Studies that reported results on race/ethnicity analysis were found to have different conclusions, given the many possible definitions used and data collection methods [23, 24]. We believe our finding, specifically, might reflect sociocultural and economic differences within our population, affecting mainly access to specialized medical care and health care in general.

Sex is another predictor with heterogeneous results in the literature. Many have not shown significant difference in the risk of death between men and women with PD [20, 25, 26]. Some studies have shown increased risk among men [4, 24], while others pointed a greater risk among women [6]. We found a higher risk of death among men. However, this result must be taken with caution, due to the statistical reasons mentioned above (see Results). Further research is needed to estimate the real effect of this variable since the proportional hazards assumption was not met.

Motor subtypes have been constantly associated with different clinical courses and mortality in the literature. There is a greater motor and functional decline [27], as well as an increased mortality rate, in the PIGD subtype [3, 5]. We found no difference in mortality risk between subtypes. This might be explained by short follow-up time. It is important to remember that motor features may change over time. We considered the motor subtype in the first evaluation. Furthermore, motor progression and functional decline were not evaluated, what could have been an important outcome in our cohort.

Age at onset and age at baseline were strong predictors of mortality, as was the case in various studies. A recent systematic review and meta-analysis found the only independent predictors of mortality were those two variables [1]. In our study, age at first visit, per year increase, had a HR of 1.05 (95% CI 1.02–1.08, *p* = 0.001). Onset before age 60 years was associated with a 70% increase in chance of survival. However, age at onset and chronological age still need to be further evaluated by inception cohorts, preferentially community-based, and with longer follow-ups in our population to confirm these findings.

Our study has limitations. The study cohort consisted only of prevalent cases at the hospital level, and with different disease durations at first evaluation. This type of cohort is particularly vulnerable to survival bias due to selection bias. All patients were referred to a specialized movement disorders clinic, so findings might not be representative of the general population. Age at onset and consequently disease duration were collected from self-reporting, thus subjected to measurement error. However, this method is widely used in the literature, including those studies reporting survival in PD. Despite these limitations, our study still holds descriptive value and, to some extent, clinical prognostic value.

Finally, we feel that PD should be viewed not as a single pathological entity, but rather as a clinical diagnosis with different pathophysiologic and genetic basis, clinical phenotypes, and prognosis. As such, treatment should be tailored to each of these subtypes, and different populations must be evaluated to allow individualized therapeutic approaches. Although we did not find difference in mortality hazards between motor subtypes in our study, we believe they do exist and plan to expand our sample size and follow-up time to continue to explore this factor.

## Supplementary Material

Supplementary figure: Survival Curves According to Gender.
We show the relationship between gender and mortality. Women may have a higher hazard at baseline up to 12 years of disease duration, when men show a higher hazard. However, this results must be taken with caution, given the low number of patients with disease duration over 12 years.

## Figures and Tables

**Table 1:** Clinical characteristics of patients with PD at the time of the first evaluation.

Characteristic	All patients	Patients alive at last follow-up	Patients who died
*N*	206	163	43
Age at onset, mean (sd) years	57.9 (11.5)	56.3 (0.89)	63.7 (1.54)^*∗∗∗*^
Age at onset, *n* (%)			
<60 years	113 (55%)	99 (61%)	14 (33%)^*∗∗∗*^
≥60 years	93 (45%)	64 (39%)	29 (67%)^*∗∗∗*^
Disease duration, mean (sd) years	8.32 (4.99)	8.36 (4.97)	8.16 (5.16)
Range	1–29	1–29	1–25
Gender, *n* (%)			
Female	105 (51%)	88 (54%)	17 (40%)^*∗*^
Male	101 (49%)	75 (46%)	26 (60%)^*∗*^
Race, *n* (%)			
White	182 (89%)	148 (91%)	34 (79%)^*∗∗*^
Nonwhite	23 (11%)	14 (9%)	9 (21%)^*∗∗*^
Clinical subtype, *n* (%)			
PIGD	108 (55%)	85 (56%)	23 (56%)
Mixed	22 (11%)	18 (12%)	4 (9%)
Tremor	65 (33%)	49 (32%)	16 (37%)
Dementia, *n* (%)	51 (29%)	35 (25%)	16 (47%)^*∗∗*^
Depression	64 (31%)	51 (31%)	13 (30%)
Hoehn&Yahr, mean (sd)	2.7 (0.81)	2.7 (0.82)	2.7 (0.78)
Schwab-England's ADL, mean (sd)	73 (22.8)	73.5 (22.3)	71.9 (24.9)

*Note*. ^*∗*^ *p* < 0.10; ^*∗∗*^ *p* < 0.05; ^*∗∗∗*^ *p* < 0.01. PIGD: postural instability-gait disorder phenotype. Dementia was defined as Minimental State Examination score lower than or equal to 23 (maximum score: 30). ADL: activities of daily living. Percentages calculated out of those with information.

**Table 2:** Comorbidities and risk factors among PD patients.

Comorbidities, *n* (%)	All patients *n* = 206	Patients alive at last follow-up *n* = 163	Patients who died *n* = 43
CVD Risk factors			
Smoking	38 (19%)	31 (19%)	7 (16%)
Dyslipidemia	20 (10%)	17 (10%)	3 (7%)
Hypertension	101 (49%)	85 (52%)	16 (37%)^*∗*^
Diabetes	29 (14%)	23 (14%)	6 (14%)
Any risk factor	124 (60%)	103 (64%)	21 (49%)^*∗*^
Major comorbidities			
Heart disease	29 (14%)	22 (14%)	7 (16%)
Stroke	9 (4%)	8 (5%)	1 (2%)
Lung disease	13 (6%)	10 (6%)	3 (7%)
Kidney failure	6 (3%)	4 (2%)	2 (5%)
Cancer	21 (10%)	16 (10%)	5 (12%)
Any major comorbidity	67 (33%)	52 (33%)	15 (35%)
Psychiatric			
Depression	64 (31%)	51 (31%)	13 (30%)

*Note*. ^*∗*^ *p* < 0.10. CVD: cardiovascular disease, smoking: past and current smoking.

**Table 3:** Causes of death among PD patients.

Causes of death *n* (%)	Patients who died *n* = 43
Pneumonia	12 (28%)
CVD	8 (19%)
Cancer	6 (14%)
Other infections	5 (12%)
COPD	4 (9%)
Parkinson	3 (7%)
Other causes	5 (11%)

*Note*. CVD: cardiovascular disease, COPD: chronic obstructive pulmonary disease.

**Table 4:** Association between baseline characteristics and mortality, expressed as mortality hazard ratio with 95% confidence interval (CI).

	Univariate models		Adjusted model	
	HR (95% CI)	*p* value	HR (95% CI)	*p* value
Age of onset, *per year increase*	1.06 (1.03–1.09)	<0.001	1.06 (1.02–1.10)	0.004
Age at onset <60 years	0.32 (0.16–0.64)	0.001	—	—
Age at first visit, *per year increase*	1.05 (1.02–1.08)	0.001	—	—
Disease duration	0.87 (0.74–1.02)	0.093	0.86 (0.70–1.07)	NS
Female^*∗*^	11.2 (0.89–140.9)	0.062	76.5 (2.03–2886)	0.019
Race^*∗∗*^	2.11 (1.00–4.43)	0.049	3.41 (1.21–9.58)	0.021
Subtype PIGD^*∗∗∗*^	0.82 (0.43–1.56)	NS	0.83 (0.22–3.17)	NS
Mixed	0.67 (0.22–2.02)	NS	1.31 (0.47–3.67)	NS
Dementia	1.99 (1.01–3.92)	0.046	1.8 (0.80–4.01)	NS
Depression	1.00 (0.52–1.94)	NS	0.88 (0.36–2.14)	NS
Hoehn&Yahr	0.90 (0.62–1.32)	NS	1.05 (0.53–2.06)	NS
ADL	1.00 (0.99–1.01)	NS	1.00 (0.98–1.02)	NS
Any risk factor	0.60 (0.33–1.12)	NS	0.37 (0.16–0.84)	0.018
Any major comorbidity	1.00 (0.54–1.89)	NS	1.62 (0.63–4.16)	NS

*Note.* Univariate models include only one variable at a time. The adjusted model included all listed covariates simultaneously. Indicator variable for age of onset lower than 60 years and age of first visit are not included in the adjusted model because of collinearity. ^*∗*^Female: time-varying component estimated because of violation of proportional hazards assumption. ^*∗∗*^Race: nonwhites as compared to whites. ^*∗∗∗*^Subtype: reference group: tremor. PIGD: postural instability-gait disorder subtype. ADL: activities of daily living. NS: nonstatistically significant, *p* > 0.10.
