# Supplementary material for: Clinical and Epidemiological Factors Associated with Mortality in Parkinson's Disease in a Brazilian Cohort
Source: Parkinsons Dis. 2015 Dec 27;2015:959304. doi: 10.1155/2015/959304 (PMC4706959; doi:10.1155/2015/959304)
Supplement: Supplementary file 1 — Supplementary figure: Survival Curves According to Gender. We show the relationship between gender and mortality. Women may have a higher hazard at baseline up to 12 years of disease duration, when men show a higher hazard. However, this results must be taken with caution, given the low number of patients with disease duration over 12 years. [file 959304.f1.docx]

**Supplemental Figure 1. Survival Curves According to Gender**
